# Associations of C‐reactive protein and homocysteine with neuropsychological outcomes in older adults at risk of dementia

**DOI:** 10.1002/alz.083882

**Published:** 2025-01-09

**Authors:** Rachael Yu, Johannes C Michaelian, Shawn Dexiao Kong, Catriona Ireland, Kimberley Bassett, Sharon L Naismith

**Affiliations:** ^1^ The University of Sydney, Camperdown, NSW Australia; ^2^ Healthy Brain Ageing Program, Brain and Mind Centre, University of Sydney, Camperdown, NSW Australia; ^3^ The University of Sydney, Sydney, NSW Australia

## Abstract

**Background:**

Inflammation is becoming increasingly recognised as a core feature of dementia and neurodegenerative processes. High‐sensitivity C‐reactive protein (hs‐CRP) and homocysteine are blood‐based markers of non‐specific inflammation used in clinical settings. This study aimed to 1) investigate the associations of hs‐CRP and homocysteine on neuropsychological performance (i.e. verbal memory, executive function, processing speed) in older adults attending a memory clinic; and 2) examine whether these associations differed for individuals at varying risk stages of dementia, i.e. with subjective cognitive decline (SCD) vs. mild cognitive impairment (MCI).

**Method:**

We recruited older adults aged ≥ 50 years with new‐onset cognitive concerns without dementia. A fasting blood sample was used to obtain concentrations of serum hs‐CRP (mg/L) and plasma homocysteine (µmol/L). Hs‐CRP levels were grouped into low <1.0mg/L, moderate 1.0‐3.0mg/L, and high risk >3.0‐10.0 mg/L. A standardised neuropsychological assessment was conducted by a clinical neuropsychologist, and composite scores for each neuropsychological outcome were calculated as an average of z‐scores of all tests within the same domain. Classifications of MCI were determined by clinician consensus as having objective impairments in neuropsychological tests (≥1.5SDs below normative scores). Multiple regression analyses were conducted, adjusting for relevant demographic and clinical factors.

**Result:**

The final sample consisted of 423 participants (Mean age = 67.37 years [SD = 8.15], 63.1% female, mean MMSE = 28.95 [SD = 1.35]), who were classified as either having SCD (n = 158) or MCI (n = 265). Hs‐CRP and homocysteine concentrations did not differ between SCD and MCI groups. In participants with SCD, high‐risk CRP concentrations were significantly associated with poorer executive function (β = ‐.226, 95% CI = [‐0.692, ‐0.059], p = .020) and processing speed (β = ‐.212, 95% CI = [‐0.584, ‐0.025], p = .033). There were no significant associations between CRP and neuropsychological outcomes in those with MCI, or in the total sample. Homocysteine was not associated with cognitive outcomes.

**Conclusion:**

CRP may be involved in early disruptions to cerebral frontal‐subcortical pathways. Targeting inflammation in the earliest stages of cognitive decline, where subjective complains are paramount, may be a viable strategy for prevention.

